# New Materials from the Integral Milk Kefir Grain Biomass and the Purified Kefiran: The Role of Glycerol Content on the Film’s Properties

**DOI:** 10.3390/polym16223106

**Published:** 2024-11-05

**Authors:** Yuly A. Ramírez Tapias, Guillermo D. Rezzani, Juan F. Delgado, Mercedes A. Peltzer, Andrés G. Salvay

**Affiliations:** 1 Laboratorio de Obtención, Modificación, Caracterización y Evaluación de Materiales, Departamento de Ciencia y Tecnología, Universidad Nacional de Quilmes, Roque Sáenz Peña 352, Bernal B1876BXD, Argentina; yuly.tapias@unq.edu.ar (Y.A.R.T.); guillermo.rezzani@unq.edu.ar (G.D.R.); mercedes.peltzer@unq.edu.ar (M.A.P.); 2 Consejo Nacional de Investigaciones Científicas y Técnicas (CONICET), Godoy Cruz 2290, Buenos Aires C1425FQB, Argentina

**Keywords:** polysaccharide-based films, biomass-based films, milk kefir, kefiran, plasticisation, antiplasticisation, glycerol

## Abstract

Microbial exopolymers are gaining attention as sources for the development of biodegradable materials. Milk kefir, a fermented dairy product produced by a symbiotic community of microorganisms, generates milk kefir grains as a by-product, consisting of the polysaccharide kefiran and proteins. This study develops two materials, one from whole milk kefir grains and another from purified kefiran. Film-forming dispersions were subjected to ultrasonic homogenisation and thermal treatment, yielding homogeneous dispersions. Kefiran dispersion exhibited lower pseudoplastic behaviour and higher viscous consistency, with minimal effects from glycerol. Both films exhibited continuous and homogeneous microstructures, with kefiran films being transparent and milk kefir films displaying a yellowish tint. Analysis revealed that milk kefir films comprised approximately 30% proteins and 70% kefiran. Kefiran films demonstrated stronger interpolymeric interactions, as evidenced using thermogravimetric and mechanical tests. Glycerol increased hydration while decreasing thermal stability, glass transition temperature, elastic modulus, and tensile strength in both films. However, in kefiran films, elongation at the break and water vapour permeability decreased at low glycerol content, followed by an increase at higher plasticiser contents. This suggests an unusual interaction between glycerol and kefiran in the absence of proteins. These findings underscore differences between materials derived from the whole by-product and purified kefiran, offering insights into their potential applications.

## 1. Introduction

Natural polymers have been continuously explored for applications in materials science due to their biodegradable nature and renewability, which makes them attractive in the context of the development of more environmentally friendly materials and processes [1,2,3]. Despite their hydrophilicity, which presents challenges in certain applications, biopolymeric materials derived from proteins and polysaccharides have been extensively studied. These investigations have shown them to be promising alternatives, particularly in the field of food packaging, to helping develop eco-friendly solutions [4,5,6]. Materials derived from microbial exopolysaccharides, either purified or combined with the biomass components from which they originate, have garnered significant attention due to their versatility and potential to replace conventional non-biodegradable materials [7,8,9,10]. This growing interest reflects a continuing shift towards packaging solutions that are not only functional, but are also sustainable, helping to reduce the use of traditional plastic packaging.

Plasticisers are essential for enhancing the integrity and mechanical properties of biopolymeric films [1,4,6]. Typically, plasticisers are small molecules, with glycerol—a single triol compound—being the most commonly used in biopolymer-based films due to its good miscibility and low cost [1,11]. It has been suggested that plasticisers destabilise interpolymeric hydrogen bonds, reducing intermolecular forces, and thereby increasing the mobility and the space between polymer chains [6,7,11]. Consequently, plasticisers can modify mechanical properties, such as reducing tensile strength and hardness, while increasing the elongation at break and the flexibility and fracture resistance of the polymer matrix [6,9]. In contrast, plasticisers usually increase the hydration of the film and decrease the water vapour barrier properties [7,12]. Despite these general trends, further investigation is needed to fully understand the complex interactions between biopolymers and plasticisers, which could clarify unresolved questions regarding film behaviour [11], including the mechanisms behind antiplasticisation [13,14,15].

Exopolysaccharides are extracellular polysaccharides produced by many bacteria and secreted by specific membrane proteins [16,17]. These exopolysaccharides typically have a high average molecular weight and high polydispersity, accumulating extracellularly and imparting a gelatinous appearance to the culture [17]. Milk kefir, a fermented dairy beverage produced by a symbiotic community of bacteria and yeasts, generates a by-product known as milk kefir grains, primarily consisting of kefiran exopolysaccharide with a secondary fraction of proteins [10,18]. Kefiran is a branched heteropolysaccharide, slightly yellow, water-soluble, and composed of approximately equal amounts of D-glucose and D-galactose [8,10,19,20]. It is typically produced by several *Lactobacillus* species including *L. kefiranofaciens*, *L. kefirgranum*, and *L. parakefir*, as well as by other unidentified *Lactobacillus* species [19]. These bacteria excrete kefiran polysaccharides with elevated molecular weights and polydispersity [8,18], ranging from 50 to 15,000 kDa [8]. Kefiran has attracted significant attention due to its unique properties, including rheological and mechanical behaviours, antioxidant and biocide activities, and health-promoting effects [20].

Kefiran is a neutral polysaccharide with numerous hydroxyl groups, which gives it a polar and hydrophilic character [21]. Previous research has demonstrated the capacity of kefiran to form films with promising functional properties, making it suitable for applications such as food packaging [21,22,23,24,25,26]. However, several aspects regarding the development and properties of kefiran-based films require further improvements and clarification. For instance, these films were produced from dilute kefiran dispersions of 1 wt% [21,22] and 2 wt% [23,24,25,26], which may lead to excessively thin films or high drying energy demands [27]. Furthermore, kefiran-based films have been reported to exhibit unexpected behaviours, such as a decrease in water vapour permeability with the addition of glycerol [22] and an increase in elastic modulus at low glycerol concentrations [26]. Moreover, the question of utilising the whole biomass of milk kefir grains for film production remains unexplored. This is relevant, as using the integral by-product of fermentations for film production could prove to be cost-effective, eliminating the need for separation and purification, and may result in films with remarkable physical-chemical properties and the retention capacity of natural bioactive substances [7,9,10].

To address these challenges, this study aimed to develop films using both the integral biomass of milk kefir grains and purified kefiran. The films were obtained from film-forming dispersions containing 5 wt% of milk kefir grains and 5 wt% kefiran, which were subjected to sequential physical treatments including ultrasonic homogenisation and thermal processing. To study the role of the plasticiser, both types of films were produced with varying glycerol concentrations. The rheological properties of the film-forming dispersions were studied, and the resulting films were characterised in terms of colour, microstructure, spectroscopy, thermal properties, mechanical strength, hydration, and water vapour permeability.

## 2. Materials and Methods

### 2.1. Materials

Milk kefir grains LOMCEM SMK1 were acquired from a household in La Plata, Argentina, and stored frozen at −20 °C. Homogenised commercial milk with a standardised fat content of 3% (Ilolay, Santa Fe, Argentina) and pharmaceutical-grade ethanol 96% *v*/*v* (Bialcohol, Córdoba, Argentina) was purchased at a local market. Silica gel, analytical grade glycerol, and analytical-grade salts used for the preparation of saturated solutions were acquired from Biopack (Zárate, Argentina). Kjeldahl and thin-layer chromatography (TLC) reagents were obtained from Sigma (St. Louis, MO, USA).

### 2.2. Milk Kefir Grains and Culture Conditions

Milk kefir grains were reactivated through successive subcultures at 22 °C in 1 L of milk cultivation medium containing around 100 g of kefir grains. The medium was exchanged daily with fresh culture medium to sustain grain viability. This process was repeated multiple times to increase the biomass of milk kefir grain, resulting in a fivefold increase after ten subcultures. For film preparation and kefiran purification, the grains were separated from the fermented liquid by filtration using a plastic sieve, and were washed five times by immersion in 2 L of distilled water. The grains were then lightly pressed to remove excess water. The dry matter (d.m.) content of the washed and pressed milk kefir grains was 0.15 g per g, determined by drying at 105 °C. The protein content in the dried milk kefir grains was 30 ± 1%, measured with the Kjeldahl method [28] using a Kjeltec^®^ 8100 distillation module (Foss, Hillerød, Denmark) coupled with a DT2508 digestor module and a SR210 scrubber (Foss, Hillerød, Denmark).

### 2.3. Kefiran Isolation and Purification

Kefiran was extracted from the washed and pressed milk kefir grains, and was purified using a procedure based on the method developed by Rimada and Abraham [29], with slight modifications. Briefly, the washed and pressed milk kefir grains were treated in boiling water at a ratio of 1:10 *w*/*w* for 45 min with continuous stirring. The mixture was then cooled and centrifuged (Avanti J-26 XP centrifuge, Beckman, Brea, CA, USA) at 10,000× *g* for 20 min at 20 °C to precipitate microbial cells and proteins. The supernatant was collected, and two volumes of ethanol cooled to −20 °C were added to precipitate the polysaccharide. The entire mixture was stored overnight at −20 °C. Then, the precipitated polysaccharide was separated by centrifugation at 10,000× *g* and 4 °C for 20 min. The resulting pellets were dissolved in hot distilled water, and the precipitation procedure was repeated twice. Finally, the precipitate was dissolved in hot distilled water, and the resulting kefiran solution was freeze-dried. The absence of other simple sugars (mono or di saccharides) in the samples was confirmed using qualitative thin-layer chromatography (TLC) following the methodology published by Piermaria et al. [18]. The protein concentration in the freeze-dried kefiran, determined using the Kjeldahl method [28], was found to be less than 0.1%.

### 2.4. Preparation of Film-Forming Dispersions of Milk Kefir and Kefiran

The washed and pressed milk kefir grains were used to prepare a dispersion containing 5 wt% d.m. in distilled water. The procedure for obtaining the milk kefir film-forming dispersion was similar to that previously developed in our laboratory for the production of water kefir film-forming dispersions [7,30]. Briefly, the dispersion was subjected to high-speed homogenisation at 18,000 rpm for 5 min using an Ultraturrax T-25 device (IKA Works, Inc., Staufen, Germany) to disrupt the grain structure. This was followed by ultrasonic homogenisation at 80 W with an Ultrasonic processor VCX-750 (Sonics and Materials, Inc., Newtown, CT, USA) for 15 min, using cycles of 30 s of pulsing and 30 s of rest to ensure complete disintegration of the grains. Thermal treatment at 90 °C in a water bath was then applied for 20 min to unfold the biopolymers and deactivate any residual enzymes and microorganisms. Following this, the dispersion underwent a second high-speed homogenisation at 15,000 rpm for 1 min to break down any aggregates formed during the thermal treatment. Finally, a second ultrasonic homogenisation was performed under the same conditions as the first to ensure the production of a fine dispersion.

The freeze-dried kefiran was used to prepare a dispersion containing 5 wt% d.m. in distilled water. This dispersion was subjected to the same ultrasound–temperature–ultrasound treatments as the milk kefir dispersion to obtain the kefiran film-forming dispersion.

Both film-forming dispersions were exposed to a complete degassing process for 30 min using a vacuum pump. Subsequently, pure glycerol was added to dispersions at levels of 0, 10, 20, and 30 wt% with respect to d.m., followed by stirring for 15 min.

### 2.5. Preparation of Milk Kefir Films and Kefiran Films

To achieve films with a thickness of approximately 0.15 mm, 20 g of the film-forming dispersion were placed in each plastic Petri dish of 86 mm in diameter. Water evaporation was performed at 40 °C and 40% relative humidity (r.h.) by casting in a ventilated oven (Sanyo MOV 212F, Moriguchi, Japan) until the water content in the films reached between 10 and 15%, a process that took around 12 h. Subsequently, the films were stored at 22 °C and 43% r.h. For experimental purposes, the films were then equilibrated at 22 °C in desiccators at various r.h. levels, achieved using saturated solutions of NaOH, MgCl_2_, K_2_CO_3_, Mg(NO_3_)_2_, NaBr, NaCl, and BaCl_2_, to generate atmospheres of 10, 33, 43, 52, 57, 75, and 90% r.h., respectively. Dried atmospheres were created using silica gel.

### 2.6. Characterisation

#### 2.6.1. Rotational Rheology Measurements of the Film-Forming Dispersions

Flow curves were acquired through rotational experiments utilising an AR-G2 rheometer (TA Instruments, New Castle, DE, USA) fitted with a 2° steel cone geometry (40 mm in diameter and 55 μm of truncation gap). Measurements were conducted in triplicate at 22 °C using 0.6 mL of film-forming dispersions, with shear rates ranging from 0.005 to 1000 s^−1^. Shear stress *τ* (Pa) as a function of shear rate *γ* (s^−1^) was recorded, and the resulting curves were fitted with the Herschel–Bulkley model as described in Equation (1):

(1) $$\tau =\tau_{0}+K\;\gamma^{n}$$

where $\tau_{0}$ (Pa) is the yield stress, representing the maximum value of *τ* for a strain rate equal to zero, *K* is the fluid consistency index, linked to the apparent viscosity of the dispersion, and *n* is the flow behaviour index indicating the deviation to Newtonian flow type (*n* > 1 for dilatant and *n* < 1 for pseudoplastic fluids). The experimental data were fitted using OriginPro 8 (OriginLab Corporation, Northampton, MA, USA).

#### 2.6.2. Visual Appearance, Quality Evaluation, Thickness Measurements, and Density of the Films

The visual examination of the films was conducted using a Samsung SM-A145M camera, capturing photographs of the sample surfaces from a height of 20 cm under natural light. The quality assessment of the films, focused on handleability, homogeneity, and continuity, was evaluated in three independent replicates [30,31]. The thickness of the films was measured with a digital calliper (±10^−6^ m; 3109-25-E, Insize Co., Suzhou, China). Measurements were taken at 20 distinct positions on each film, resulting in an average thickness of around 0.15 mm per specimen. For density determination of the dried films, circular samples with an area of 58 cm^2^ were dehydrated in containers with silica gel for 10 days. The films were then weighed using an analytical balance (±10^−4^ g). The density of the dried film *ρ_d.f._* (g m^−3^) was calculated using Equation (2):

(2) $$\rho_{d.f.}=m/\left(AL\right)$$

where *m* is the dry mass (g), *A* the area (m^2^), and *L* is the thickness (m).

#### 2.6.3. Colour Determination (CIELab Coordinates) of the Films

The colour of the samples was assessed with a Konica Minolta CR-400 colourimeter (Tuscaloosa, AL, USA) using illuminant C and a 2° observer following the CIE 1931 standard [32]. The films were placed on a white background, and the CIELab colour space was utilised to determine the parameters L* (lightness), a* (green to red), and b* (blue to yellow). The total colour change ∆*E* was calculated using Equation (3):

(3) $$\Delta E=\sqrt{{\left(L^{*}-L_{0}\right)}^{2}+{\left(a^{*}-a_{0}\right)}^{2}+{\left(b^{*}-b_{0}\right)}^{2}}$$

where *L*_0_, *a*_0_, and *b*_0_ represent the coordinates corresponding to the unplasticised kefiran film, considered the reference to determine the colour change. Measurements were taken at five different points for each formulation.

#### 2.6.4. Microstructural Characterisation Using Scanning Electron Microscopy (SEM)

The microstructure of the films was investigated by observing the surfaces and cross-sections using a scanning electron microscope FEI-Quanta 200 (Fei Co., Hillsboro, OR, USA) operated at 15 kV. Cross-sections were prepared by cutting the samples at −20 °C with a sharp blade. Subsequently, all of the samples were placed in the specimen holder and stored at 22 °C and 43% r.h. To enhance visibility under the microscope, the samples were coated with a layer of gold. Images of the surfaces (magnification 3000×) and cross-sections (magnification 1000× and 20,000×) of the films were acquired under high-vacuum conditions.

#### 2.6.5. Attenuated Total Reflectance-Fourier Transform Infrared (ATR-FTIR) Spectroscopy Analyses of the Films

Infrared spectra of films were recorded in the range of 4000–500 cm^−1^ using a Fourier-Transform Infrared Analyser (FTIR) Shimadzu IR-Affinity (Shimadzu Co., Kyoto, Japan) equipped with an attenuated total reflectance monolithic diamond crystal module (GladiATR, Pike Technologies, Madison, WI, USA). The spectra were obtained by averaging 48 scans with a resolution of 4.0 cm^−1^ and Happ-Genzel apodisation. A blank spectrum was recorded before each test to account for humidity and carbon dioxide in the air by subtracting their effects from the spectra. Each measurement was performed in triplicate.

#### 2.6.6. Thermogravimetric Analyses (TGA) of the Films

To investigate the thermal degradation of the films, the weight loss of the samples as a function of temperature was recorded using a TA Instruments Q-500 thermogravimetric analyser (New Castle, DE, USA). Before the experiment, the samples were equilibrated at 22 °C and 52% r.h. Approximately 3 mg of each sample was placed in a platinum sample pan and heated from 40 to 800 °C at a rate of 10 °C per minute. The experiments were carried out in triplicate under a nitrogen atmosphere with a flow rate of 60 mL per minute. The initial degradation temperature (*T*_0_) was defined as the temperature at which 15% of the weight had been lost. The temperature corresponding to the maximum degradation rate (*T*_max_) was obtained from the peak of the derivative of the weight loss with respect to temperature using the TA Universal Analysis software (v4.5, TA Instruments, New Castle, DE, USA).

#### 2.6.7. Differential Scanning Calorimetry (DSC) Studies of the Films

The thermal behaviour of films under controlled heating was analysed using a differential scanning calorimeter (TA Instruments Q200, New Castle, DE, USA). Film samples were placed into Tzero^®^ aluminium pans (TA Instruments, New Castle, DE, USA) and dehydrated at 22 °C in containers with silica gel for 10 days. Subsequently, the pans were rapidly sealed with hermetic lids, each containing approximately 3 mg of dried samples. Thermograms were obtained within a range from −80 °C to 180 °C, with an initial equilibration step at −80 °C for 1 min, followed by a temperature increase at a rate of 10 °C per minute. Glass transition temperatures (*T*_g_) were determined at the midpoint using the TA Universal Analysis software (v4.5, TA Instruments, New Castle, DE, USA). The experiments were conducted in triplicate.

#### 2.6.8. Mechanical Uniaxial Tensile Experiments of the Films

Uniaxial tensile tests of the films were performed using a Universal Testing Machine (TC-500 II-Series, Micrometric, Buenos Aires, Argentina) equipped with a 300 N cell. Rectangular samples of 50 mm in length and 10 mm in width were prepared. The samples were equilibrated in an atmosphere at 52% of r.h. and then placed between the machine’s jaws with an effective gauge length of 25 mm. The testing speed was set at 5 mm per minute, and ten specimens of each composition were tested at 22 °C. Elongation at break (*e*_%_, %), maximum tensile strength (*TS*_max,_ MPa), and elastic modulus (*Y*, MPa) were calculated from the resulting stress–strain curves, with averages taken from ten replicates, following the guidelines of ASTM D882 1997 [33].

#### 2.6.9. Water Sorption Isotherms of the Films

Water sorption isotherms were determined gravimetrically at 22 °C, following the standard procedure previously described [20]. Dried film samples, each with a superficial area of 58 cm^2^, were placed in 3 L desiccators at different water activities (*a_w_*). The samples were periodically weighed using an analytical balance (±10^−4^ g), and the moisture content was monitored at each condition until a constant weight was achieved. The water content, or hydration (*h*), was expressed in units of g of water per g of d.m. and was evaluated as a function of *a_w_* (*a_w_* = % r.h./100). Experiments were conducted in triplicate, and the resulting isotherms were fitted using the Guggenheim–Anderson–De Boer (GAB) model [34], as shown in Equation (4):

(4) $$h\left(a_{w}\right)=\frac{Ncka_{w}}{\left[\left(1+\left(c-1\right)ka_{w}\right)\left(1-ka_{w}\right)\right]}$$

where *N* represents the monolayer water content (g of water per g of d.m.), which is associated with the primary binding sites of hydration water molecules; *c* is a parameter related to the sorption heat of the monolayer, reflecting the binding force of water to the monolayer; while *k* is related to sorption heat multilayer, which is linked to the ability of water to bind to the multilayer. For each sample formulation, one water sorption isotherm was obtained, and the water content *h* corresponding to each *a_w_* was determined as the mean of three experimental measurements, including their respective errors. The values of the parameters *N*, *c*, and *k*, along with their associated errors, were derived by fitting the experimental data points to the GAB model using OriginPro 8 (OriginLab Corporation, Northampton, MA, USA).

#### 2.6.10. Experimental Water Vapour Permeability Measurements

The experimental water vapour permeability (*P_w_^exp^*) of the films was determined using the cup method described in ASTM-E96 2016 [35], with some modifications [12]. The films were sealed on the top of cups containing a saturated salt solution of BaCl_2_, providing a high r.h. of 90%. The test cups were placed in 7 L desiccators maintained at a constant temperature of 22 °C and 10% r.h. To ensure uniform conditions inside the desiccators and over the films, a fan was installed, as previously recommended [36]. The weight loss of the test cups, indicative of water vapour transport through the film, was monitored using an analytical balance (±10^−3^ g). Weight loss *m* (g) versus time *t* (min) was plotted, and once the steady state (indicated by a linear trend) was established, data were recorded for 40 h further. *P_w_^exp^* was determined as displayed in Equation (5):

(5) $$P_{w}^{exp}=\left(\frac{1}{A}\frac{\Delta m}{\Delta t}\right)\frac{L}{\Delta p_{w}}$$

where *A* = 2.2 ×10^−3^ m^2^ is the effective area of the exposed film, Δ*m*/Δt denotes the slope of a linear regression of the weight loss versus time, *L* (m) is the film thickness, Δ*p_w_* = (*p_w_*_2_ − *p_w_*_1_) is the differential water vapour pressure across the film, and *p_w_*_2_ and *p_w_*_1_ are the partial pressures (Pa) of water vapour at the film surfaces inside and outside the cup, respectively [37]. *P_w_^exp^* is given in units of g s^−1^ m^−1^ Pa^−1^. Experiments were performed in triplicate.

#### 2.6.11. Determination of Effective Water Solubility and Effective Water Diffusion in the Films

When there are no pores, faults, or punctures in the film, *P_w_^exp^* can be expressed as Equation (6) [38,39]:

(6) $$P_{w}^{exp}=D_{w}^{eff}S_{w}^{eff}$$

where *S_w_^eff^* (g m^−3^ Pa^−1^) is the effective water solubility coefficient over the concentration range *c_w_*_2_ to *c_w_*_1_, corresponding to water vapour pressures *p_w_*_2_ and *p_w_*_1_, respectively, and *D_w_^eff^* (m^2^ s^−1^) is the effective diffusion coefficient over the water concentration range *c_w_*_2_ to *c_w_*_1_. Water sorption isotherms were used to evaluate the water concentration *c_w_* (*c_w_* = *h*(*a_w_*) × *ρ_d.f._*) of each film specimen surface in the permeability experiment. *S_w_^eff^*, corresponding to the pressure gradient or *a_w_* interval [*a_w_*_2_ = 0.9; *a_w_*_1_ = 0.1], was obtained using Equation (7) [37]:

(7) $$S_{w}^{eff}=\left[\left(h\left(a_{w2}\right)-h\left(a_{w1}\right)\right)/\left(p_{w2}-p_{w1}\right)\right]\times \rho_{d.f.}$$

where *h*(*a_w_*_2_) and *h*(*a_w_*_1_) are the water content of the film (g H_2_O per g d.m.) at its underside surface at *p_w_*_2_ and its surface outside the cup at *p_w_*_1_, respectively. According to Equation (6), *D_w_^eff^* was then calculated using Equation (8):

(8) $$D_{w}^{eff}=P_{w}^{exp}/S_{w}^{eff}$$

### 2.7. Statistical Analyses

Statistical analyses were performed using OriginPro 8 (OriginLab Corporation) and R software (version 3.4.4, R Foundation for Statistical Computing, Vienna, Austria). All results are expressed as means with standard deviation. An analysis of variance was performed on the data, and a post hoc test (Tukey HSD) was used to compare the means. Differences were deemed significant at *p* < 0.05. The errors in the parameters from the Herschel–Bulkley and GAB models, derived from the flow curves and water sorption isotherms, respectively, were estimated using a fit analysis.

## 3. Results and Discussion

### 3.1. Rotational Rheology of Film-Forming Dispersions

**Figure 1:**
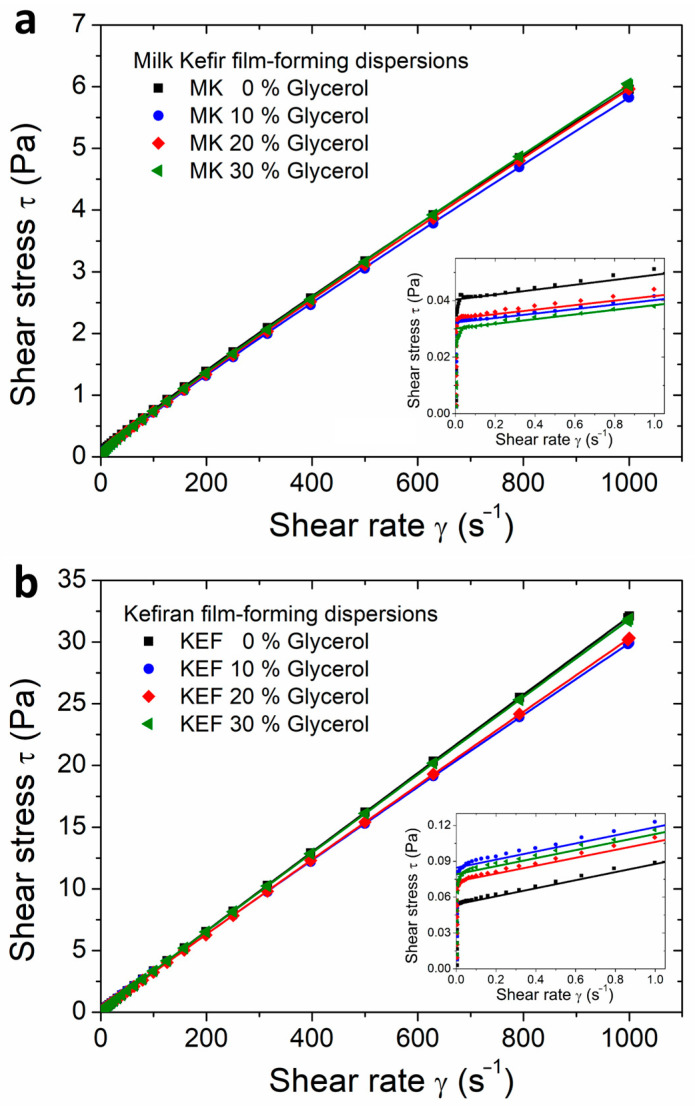
Rotational rheology of film-forming dispersions with different contents of glycerol. (**a**) Flow curves of milk kefir dispersions. (**b**) Flow curves of kefiran dispersions. Flow curves were fitted using the Herschel–Bulkley model (Equation (1)). Fitted parameters are shown in Table 1. The low-shear-rate region is shown in the insert of the figures.

Understanding the rheological properties of film-forming dispersions is crucial, as they dictate the processing conditions and machinability necessary for large-scale film production [22]. The flow behaviour of milk kefir and kefiran film-forming dispersions is shown in Figure 1. All of the studied dispersions were homogeneous, exhibiting no phase separation. The Herschel–Bulkley model (Equation (1)) was used to analyse the shear stress *τ* versus the shear rate *γ* data, and the resulting model parameters are summarised in Table 1. High values of the statistical parameter *R*^2^ indicate a good fit of the model to the experimental data.

All of the studied dispersions exhibited pseudoplastic behaviour (*n* < 1) beyond their yield stress *τ*_0_. As illustrated in Table 1, this shear-thinning behaviour was more pronounced in dispersions prepared from milk kefir grains. Milk kefir dispersions displayed significantly lower shear stress values compared to kefiran dispersions, as shown in Figure 1a,b. The consistency index *K*, directly related to the apparent viscosity, was at least three times higher for kefiran dispersions (Table 1). Additionally, kefiran dispersions exhibited higher *τ*_0_ compared to milk kefir dispersions (Figure 1 and Table 1). Fluids with *τ*_0_ > 0 display solid-like behaviour until the yield stress is surpassed, after which they transition to liquid-like behaviour when *τ* > *τ*_0_ [40]. The higher *τ*_0_ values suggest stronger interpolymeric interactions within the dispersion, consistent with observations reported in polysaccharide dispersions [30,32,41]. These results imply that the presence of proteins in milk kefir samples weakens the interactions between kefiran chains, leading to dispersions with a lower consistency index and yield stress.

As observed in Table 1, the incorporation of glycerol into the dispersions had minimal effects on the *K* and *n* parameters of both dispersions. Previous studies on 1 wt% kefiran dispersions reported that the parameters *K* and *n* remained unchanged upon the addition of glycerol at concentrations of 0, 25, and 50 wt% relative to d.m. [22]. Similarly, other researchers reported no change in the apparent viscosity for a 2 wt% kefiran dispersion after the addition of glycerol or sorbitol at 25 wt% relative to d.m. [42]. Interestingly, the effect of glycerol content on *τ*_0_ differed between milk kefir and kefiran dispersions (Table 1): *τ*_0_ increased with increasing glycerol content for kefiran dispersions, whereas it decreased for milk kefir dispersions.

A more pronounced shear-thinning behaviour (lower *n*-values) was observed in 1 wt% kefiran dispersions studied by other authors [22] compared to the kefiran dispersions investigated in the present work. However, these authors reported consistency index *K* values similar to those shown in Table 1 for 5 wt% kefiran dispersions. While *K* typically increases with polymer concentration [43], it is important to note that the 5 wt% kefiran dispersions studied in the present work were subjected to sonication. Sonication is known to break polysaccharide chains and reduce their molecular weight [44,45], thereby weakening their gelling ability and enhancing dispersibility [46]. As a result, the ultrasound treatments likely reduced *K* in the 5 wt% kefiran dispersions, bringing it in line with that of non-sonicated 1 wt% kefiran dispersions.

High viscosity in film-forming dispersions can hinder air bubble removal, resulting in film defects such as pores and holes [4,47]. Conversely, low viscosity in dilute dispersions can lead to excessively thin films or require higher drying energy [27]. This study aims to strike a balance by formulating a dispersion with a high biopolymer concentration, yet a consistency index *K* comparable to that of a low-concentration dispersion, thus minimising these issues.

### 3.2. Assessment of Quality, Visual Appearance, and Microstructure of the Films

**Figure 2:**
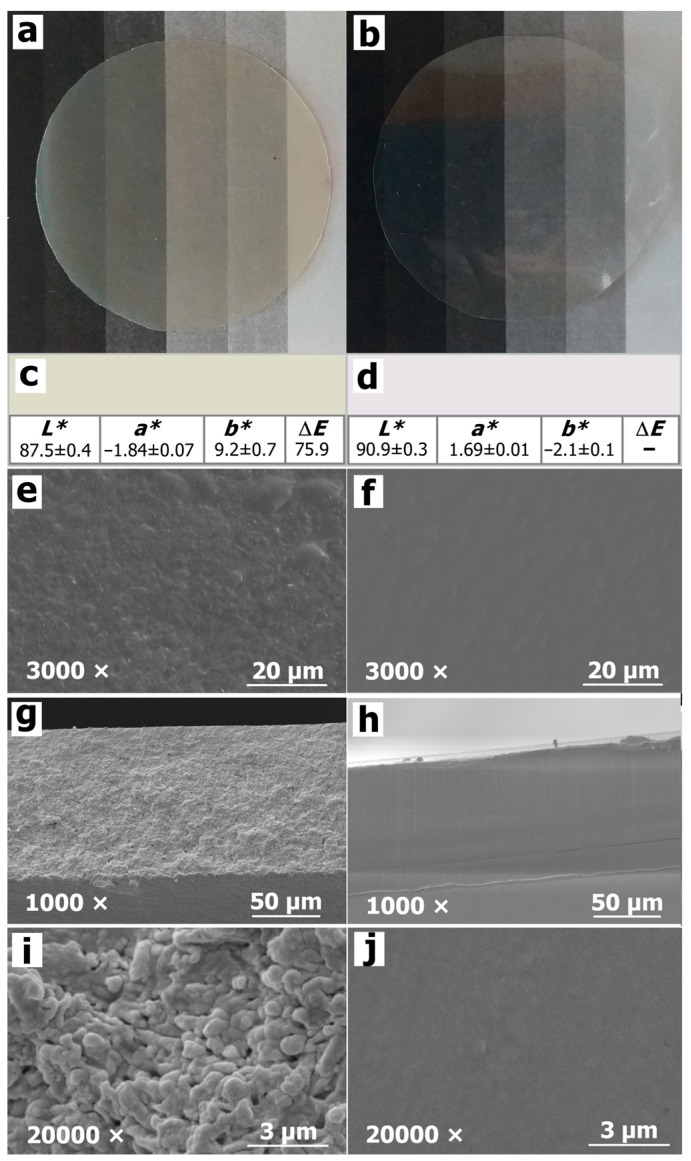
Visual appearance, colour, and microstructure of the unplasticised films. Photographs of (**a**) milk kefir film and (**b**) kefiran film; CIELab colour parameters and colour representation of (**c**) milk kefir film and (**d**) kefiran film; SEM micrographs of the surface of films at 3000× of (**e**) milk kefir film and (**f**) kefiran film; SEM micrographs of the cross-sections of films at 1000× of (**g**) milk kefir film and (**h**) kefiran film; SEM micrographs of the cross-sections of films at 20,000× of (**i**) milk kefir film and (**j**) kefiran film.

Visual inspection revealed homogeneous, continuous, and smooth films with uniform surfaces, free of air bubbles, cracks, and pores (Figure 2a,b). Plasticised films containing 10, 20, and 30 wt% of glycerol (relative to d.m.) displayed similar visual appearances compared to unplasticised films. However, film flexibility increased with rising glycerol content. Unplasticised milk kefir films were brittle, difficult to handle, and required careful removal from the Petri dish. Conversely, milk kefir films with added glycerol, as well as both unplasticised and plasticised kefiran films, were flexible and easy to peel and manipulate. This enhanced flexibility is likely due to the effective dispersion of the small glycerol molecules. It has been reported that glycerol intercalates between polymer chains, disrupting interpolymer interactions and increasing chain separation, leading to a more flexible film [7,48].

Milk kefir films displayed a yellow tint while remaining translucent (Figure 2a), whereas kefiran films exhibited high transparency (Figure 2b). Transparency and colour are crucial attributes of packaging materials, as they influence consumer perception of the product. Transparent films allow for product visibility, while coloured films could offer protection from radiation [30]. The CIELab colour parameters and colour representations for unplasticised milk kefir films and kefiran films are shown in Figure 2c,d, respectively. The addition of glycerol did not significantly alter these parameters in either film type. High L* values, particularly in kefiran films, indicated excellent clarity and transparency. Milk kefir films showed a slight reduction in L*, exhibiting a trend towards yellow (increased b* values), likely due to Maillard reactions between milk kefir proteins and carbohydrates. Milk kefir films had Δ*E* values greater than 6, indicating that the colour difference with kefiran films is perceptible to the human eye [30], which matched visual observations. Notably, the remarkable transparency of the kefiran films in this study exceeded that reported for films made from non-sonicated 2 wt% kefiran dispersions [23,24]. This suggests that sonication of the kefiran film-forming dispersion before casting resulted in a highly translucent matrix.

SEM was utilised to analyse the microstructure of the films, as shown in Figure 2e–j. The micrographs revealed continuous and homogeneous surfaces and cross-sections in both film types, devoid of pores or punctures. However, notable morphological differences were observed. Milk kefir films exhibited a rougher texture, possibly due to the presence of proteins, indicating a more rubber-like character. In contrast, kefiran films displayed a smoother surface and a more uniform structure. The concentration of glycerol did not appear to influence the microstructure as observed in SEM, indicating good compatibility between the plasticiser and both film matrices. This observation is consistent with findings from previous studies which reported no significant microstructural differences in kefiran films containing different amounts of glycerol or sorbitol [23,24]. Furthermore, X-ray diffraction analyses reported similar amorphous structures with crystallinity below 3% for both plasticised and unplasticised kefiran films [21,22]. It has been suggested that the amorphous nature of kefiran likely contributes to its excellent miscibility with glycerol within the film matrix [42].

### 3.3. Infrared Spectroscopy of the Films

**Figure 3:**
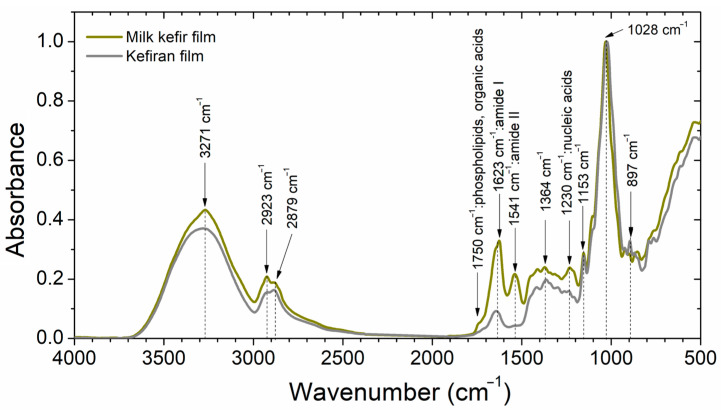
ATR-FTIR spectra of unplasticised films.

ATR-FTIR spectroscopy was employed to identify characteristic functional groups within the films. Figure 3 shows the ATR-FTIR spectra of unplasticised milk kefir film and kefiran films. For clarity, the spectra have been normalised to the major peak at 1028 cm^−1^. Both films displayed common bands, but significant differences were observed in the 1800–1200 cm^−1^ range, likely due to proteins present in the milk kefir films. Plasticisation with 10, 20, or 30 wt% glycerol resulted in similar features for both unplasticised milk kefir and kefiran films, with only increased band intensities observed. This aligns with findings for glycerol-plasticised kefiran films [21,42] and water kefir grain-based films [7], attributed to both increased water content and the presence of glycerol itself [7,8].

The kefiran spectrum shown in Figure 3 is similar to those reported for purified kefiran [26,49], kefiran films [21,42], and kefiran electrospun nanofibre materials [49]. This observation confirms the integrity of the kefiran units’ chemical structure throughout the ultrasound treatment, casting, and electrospinning processes.

Analysis of the ATR-FTIR spectra (Figure 3) revealed several key features. The broad peak at 3271 cm^−1^, assigned to the O-H stretching of water and polysaccharides [7], spanned the 3700–3000 cm^−1^ region. Milk kefir films exhibited a slightly sharper peak in this region, due to the presence of N-H bonds from proteins [30]. The bands observed at 2923 and 2879 cm^−1^ correspond to the symmetric and anti-symmetric stretching of C-H bonds in the methyl (CH₃) and methylene (CH₂) groups [21]. The differences observed between kefiran films and milk kefir films in the 3700–2700 cm^−1^ region can be attributed to the presence of proteins in milk kefir films. Similar observations have been reported in water kefir/yeast biomass blend films, suggesting that protein–polysaccharide interactions alter the chemical environment in this region, thereby affecting the stretching vibrations [30].

The most significant spectral differences were observed between 1800 and 1200 cm^−1^, primarily due to proteins in the milk kefir films. These films displayed bands at 1623 and 1541 cm^−1^, characteristic of amide I and II in proteins [30]. Additionally, peaks at 1750 and 1230 cm^−1^ were attributed to organic acids remnants of the fermentation, phospholipids, and nucleic acids from residual cell material in milk kefir films. Conversely, the kefiran film spectrum lacked these protein and cell-related peaks, but displayed a peak near 1640 cm^−1^, likely due to O-H bending vibrations in water molecules [50]. Both films exhibited small peaks and shoulders between 1400 and 1300 cm^−1^, assigned to variations in C–H bond angles [42,49].

The region between 1200 and 900 cm^−1^ displayed a prominent peak at 1028 cm^−1^ in both samples, a characteristic feature of kefiran [21]. Additional peaks corresponding to carbohydrate ring vibrations and functional groups (C–O–C, C–OH, C–H) were also detected. These peaks likely originate from the vibrational modes of glucose and galactose units within the kefiran structure, which is the primary component of milk kefir grains. Specifically, peaks at 1153 and 897 cm^−1^ were identified and assigned to vibrational modes of glucose, galactose, and β-linkages, indicative of the pure kefiran structure [21,26,51].

### 3.4. Thermogravimetric Analysis of the Films

**Figure 4:**
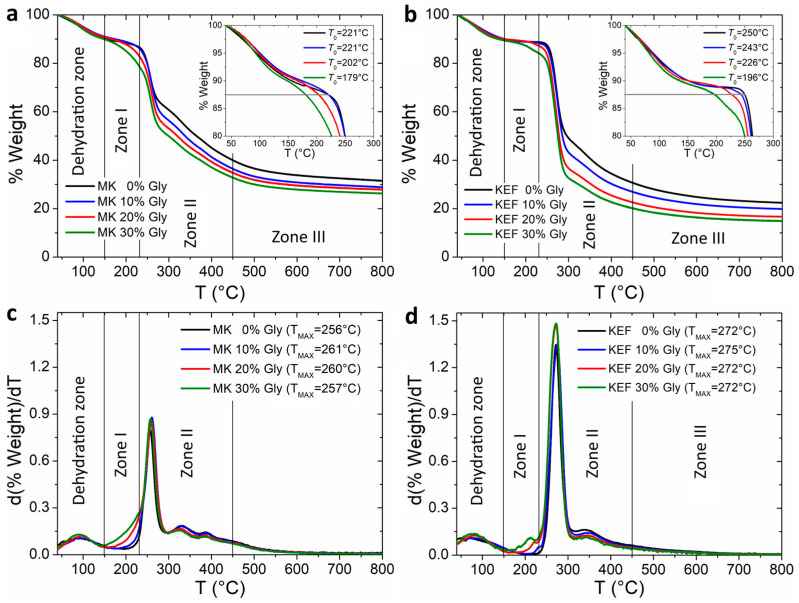
Thermogravimetric analysis of previously hydrated films at 43% r.h. (**a**) Mass loss of milk kefir (MK) films with different contents of glycerol. (**b**) Mass loss of kefiran (KEF) films with different contents of glycerol. The onset degradation temperatures (*T*_0_) are displayed in the insert of the figures. (**c**) Derivative of mass loss of milk kefir films. (**d**) Derivative of mass loss of kefiran films.

To investigate thermal degradation, a thermogravimetric analysis was conducted on films that had been previously hydrated to equilibrium at 52% r.h. Figure 4 presents the mass loss curves as a function of the temperature, revealing distinct thermal degradation zones. The initial stage, occurring below 150 °C, was associated with dehydration [30] and resulted in approximately 10% weight loss.

A primary degradation zone (Zone I) was observed between 150 °C and 230 °C. Kefiran films exhibited higher thermal stability than milk kefir films, with this trend reflected in the initial degradation temperature (*T*_0_). Glycerol incorporation reduced thermal stability in both film types. Thermal degradation in this zone was intensified with increasing glycerol content and was more pronounced for milk kefir films (Figure 4c,d). These results can be attributed to the degradation in glycerol that occurs within this temperature range [7,32] and to the onset of protein decomposition [30,52,53] present in milk kefir films.

The second degradation zone (Zone II), spanning from 230 °C to 450 °C, marked the maximum degradation rate of the samples. *T*_max_ values of 256 °C and 272 °C were determined for the unplasticised milk kefir and kefiran films, respectively (Figure 4c,d). The lower *T*_max_ of milk kefir films suggests the presence of weakened interpolymeric interactions due to the presence of proteins, which favour thermal degradation. This type of interaction between proteins and kefiran was also observed in the rheological tests of the dispersions, as discussed in Section 3.1. Additionally, the *T*_max_ value of unplasticised kefiran film in the present study was lower than the reported *T*_max_ values of 300 °C for purified kefiran [54] and 306 °C for unplasticised kefiran film obtained from a film-forming dispersion that was not sonicated [26]. It is noteworthy that the films in our study were prepared from sonicated dispersions. Ultrasound treatment is known to induce chain scission in polysaccharides [44], potentially leading to a decrease in polymer network entanglement. This reduction in entanglement density could lead to a less-rigid film matrix, thereby promoting thermal degradation and decreasing *T*_max_. On the other hand, the incorporation of glycerol did not substantially affect *T*_max_. Following *T*_max_, both samples exhibit minor and scattered peaks of thermal degradation, especially in the milk kefir films. These secondary peaks were also reported for purified kefiran [54] and kefiran films [26].

A final weight loss occurred between 450 °C and 800 °C (Zone III), attributed to the pyrolysis of carbohydrates [32,55] and massive protein degradation [30]. As illustrated in Figure 4a,b, the final residue at 800 °C was influenced by the initial glycerol content. This gradual decrease in final carbon with the glycerol content was more pronounced in kefiran films and reflects the effect of glycerol on the film’s structure. As described previously, the glycerol makes the film’s structure more susceptible to thermal degradation under dynamic conditions [7].

### 3.5. Differential Scanning Calorimetry of the Films

**Figure 5:**
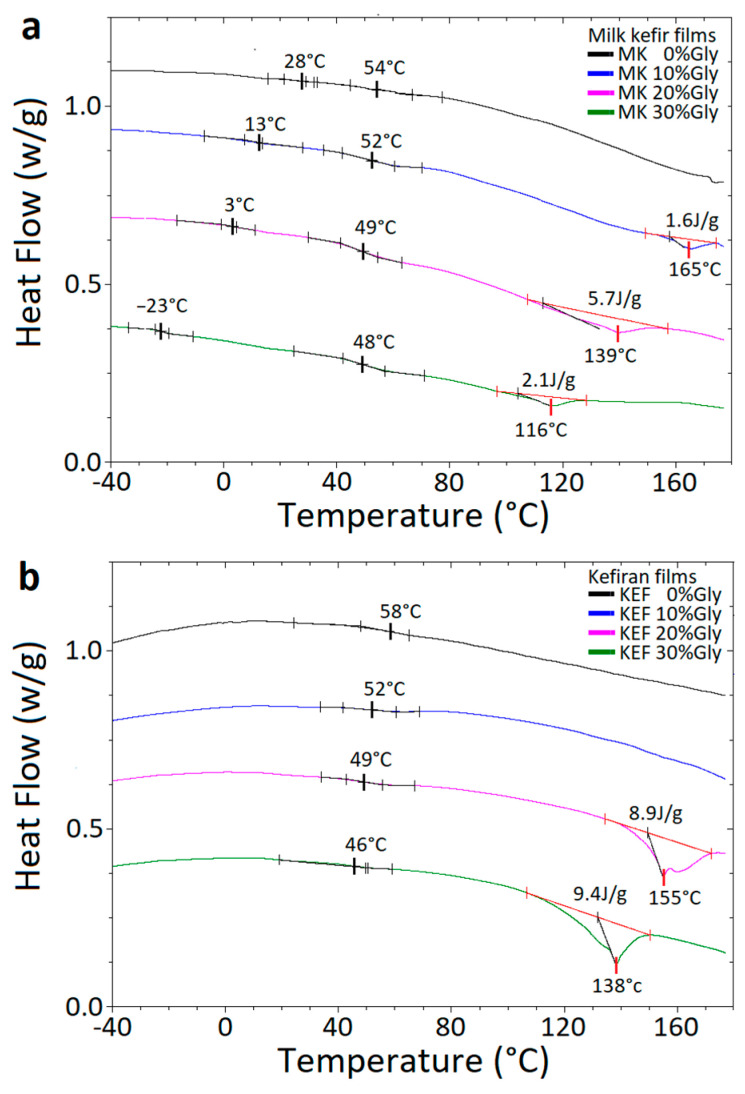
DSC thermograms and glass transition temperature of dried films with different contents of glycerol. (**a**) Milk kefir films. (**b**) Kefiran films.

To investigate thermal transitions, dehydrated films were subjected to DSC analysis. Figure 5a,b presents the DSC thermograms for milk kefir and kefiran films, respectively. The thermograms of milk kefir films show two thermal transitions for the unplasticised sample and three for the plasticised ones. All of the samples display two *T*_g_ values: one ranging from 28 to −23 °C, corresponding to the protein-rich zone of the blend, and the other from 54 to 48 °C, corresponding to the polysaccharide-rich zone. The decrease in the *T*_g_ value with glycerol addition aligns with previous studies that show a reduction in *T*_g_ when glycerol is incorporated into biopolymeric matrices [7,9,56,57]. The protein-rich zone, which is absent in kefiran films (Figure 4b), seems to be more influenced by glycerol addition. At higher temperatures, plasticised samples displayed an endotherm peak with a low heat of fusion, corresponding to the melting point of the blend. This finding is consistent with reports that kefiran has a small percentage of crystallinity [21,22]. The presence of glycerol affects this transition. It has been proposed that plasticisers reduce interpolymeric forces, increase segmental chain mobility, and improve polymer flexibility, thereby decreasing the melting point [57].

The thermograms of kefiran films (Figure 5b) confirm that the glass transition in the range from 58 °C to 46 °C corresponds to the polysaccharide fraction. In kefiran films, the addition of glycerol decreased both the *T*_g_ and the endothermic peak observed at higher temperatures. As previously described, this effect is attributed to the capacity of the glycerol to reduce hydrogen bonding within the polymer network, thereby increasing macromolecular mobility [7,57].

It is important to note that DSC experiments in the present study were performed in hermetically sealed capsules, with samples fully dehydrated prior to the analysis. Therefore, the plasticising effect of water was not expected to influence these results, unlike other studies on kefiran films [23,26]. Many previous studies have reported an endothermic peak at 84 °C for neat kefiran film and from 82 °C to 89 °C for plasticised samples [26]. Similarly, endothermic peaks have been observed at 84.7 °C (neat kefiran films) and up to 86.6 °C for γ-irradiated kefiran films [25]. The higher temperatures of endothermic peaks observed in the present study are likely due to the sample’s preparation and the conditions of the capsules used (hermetically sealed in the present work versus non-specified conditions). Therefore, it is plausible that the endothermic transitions reported in other works were influenced by the plasticising effect of water hydration and, possibly, by water evaporation during the test.

Furthermore, the *T*_g_ values of kefiran films in the present study were higher than those reported in the literature [23,26]. Ghasemlou et al. reported a *T_g_* value around 5 °C for kefiran films, which decreased to −20 °C when glycerol was added at 35 wt% relative to d.m. [23]. Montoille et al. found a *T_g_* around −20 °C for neat kefiran films, which increased to −17 °C with the addition of 5 wt% of glycerol [26]. These differences may be due to the presence of hydration water in those studies, which acts as a plasticiser, thereby lowering the *T_g_* [7,9].

### 3.6. Mechanical Properties of the Films

**Figure 6:**
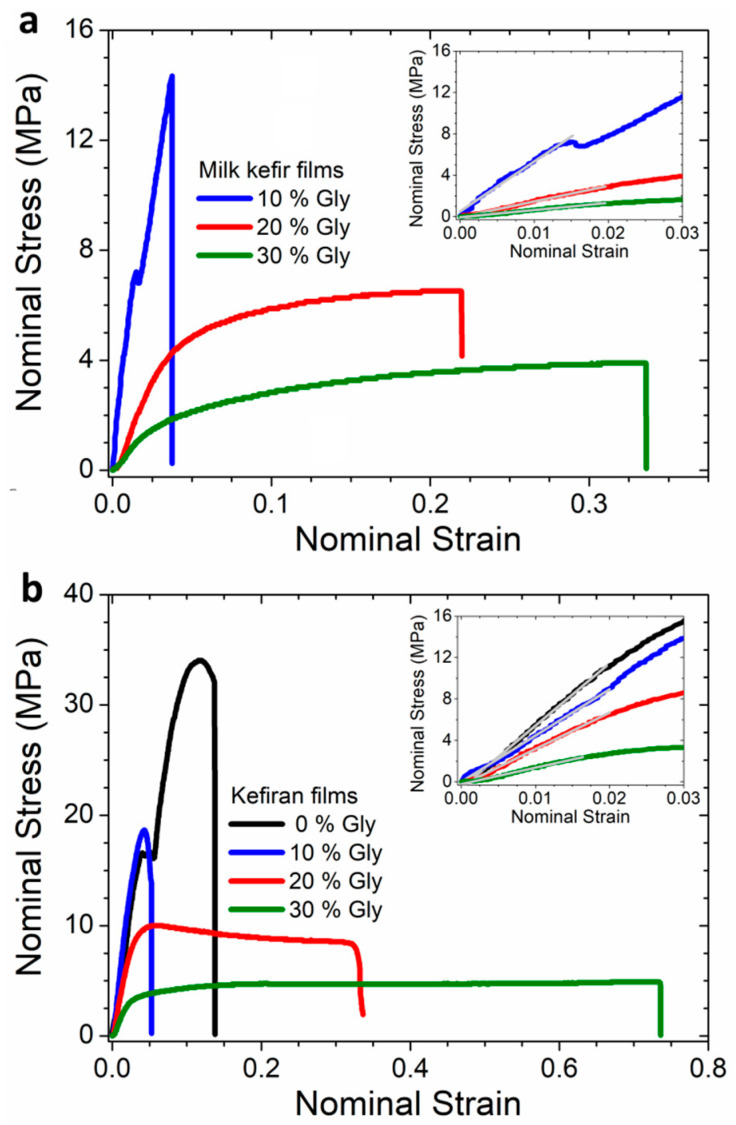
Representative stress–strain curves for one of ten replications of the mechanical test performed for films with different contents of glycerol. (**a**) Milk kefir films. (**b**) Kefiran films. Elastic modulus *Y* was calculated from the slope in the linear region (insert of the figure), maximum tensile strength *TS*_max_ from the maximum value of the nominal stress, and *e*_%_ from the maximum value of the nominal strain. Values of the mechanical parameters are shown in Table 2.

The mechanical properties of a material, specifically the elastic modulus (*Y*), maximum tensile strength (*TS*_max_), and elongation at break (*e*_%_), are determined by its composition, structure, and interpolymeric interactions. These parameters were determined from the stress–strain curves (Figure 6) and are summarised in Table 2. Unplasticised milk kefir films were too brittle to undergo testing under the specified conditions (22 °C and 52% r.h.).

Table 2 shows that increasing glycerol content reduced both *Y* and *TS*_max_ in both film types. The parameter *Y* reflects the strength of intermolecular bonds, while *TS*_max_ is associated with the quantity of these intermolecular bonds [9]. The observed reduction in *Y* and *TS*_max_ with glycerol addition in both films demonstrates the plasticiser’s role in decreasing interpolymer interactions. Conversely, glycerol addition led to a monotonic increase in *e*_%_ in milk kefir films, whereas in kefiran films, *e*_%_ initially decreased slightly at 10 wt% glycerol before increasing significantly at 20 and 30 wt% glycerol. Although biopolymeric materials typically show a correlation between the reduction in *Y* and *T*_max_ and the increase in *e*_%_ with the addition of glycerol [9,58], kefiran films displayed a non-monotonic increase in *e*_%_ response, suggesting a more complex mechanism.

Previous studies reported an unusual increase in *Y* for kefiran films at glycerol content below 5 wt% [26]. The term “antiplasticisation” has been used to describe the mechanical behaviour observed when low-molecular-weight compounds are added to certain polymer materials, leading to an increase in *Y* or a decrease in *e*_%_ [13]. Other studies demonstrated the antiplasticisation effect in the mechanical properties of kappa-carrageenan films plasticized with either glycerol or sorbitol [59]. In that work, the authors suggested that this behaviour could be due to the strong interaction of the polymer chains with the plasticiser. Accordingly, the mechanical performance observed in kefiran films suggests the presence of an antiplasticisation effect at low glycerol concentrations. These findings indicate the existence of anomalous interactions between glycerol and kefiran in the absence of proteins.

Milk kefir films displayed lower *Y* and *TS*_max_ compared to kefiran films (Figure 6, Table 2) due to the weakening of interpolymer interactions induced by the presence of proteins. This is consistent with the findings from the rheological and thermogravimetric experiments discussed in Section 3.1 and Section 3.4, respectively. Additionally, kefiran films exhibited higher *e*_%_ values.

Piermaria et al. reported values of *Y*, *TS*_max_, and *e*_%_ of ~1300 MPa, 41 ± 8 MPa, and 3 ± 1%, respectively, for unplasticised kefiran films prepared from a 1 wt% kefiran dispersion without sonication [23]. Comparable values have been obtained by other researchers for unplasticised kefiran films prepared under similar conditions [26]. A comparison of these reported values with those of the unplasticised kefiran films displayed in Table 2 suggests that the ultrasound treatment of the film-forming dispersions results in a more ductile material. This could be due to the sonication-induced breakdown of polysaccharide chains, resulting in a film matrix with reduced entanglement density. This observation agrees with the findings from thermogravimetric studies (Section 3.4) and translates to a more flexible material, potentially offering advantages for specific packaging applications.

### 3.7. Hydration Properties of the Films

**Figure 7:**
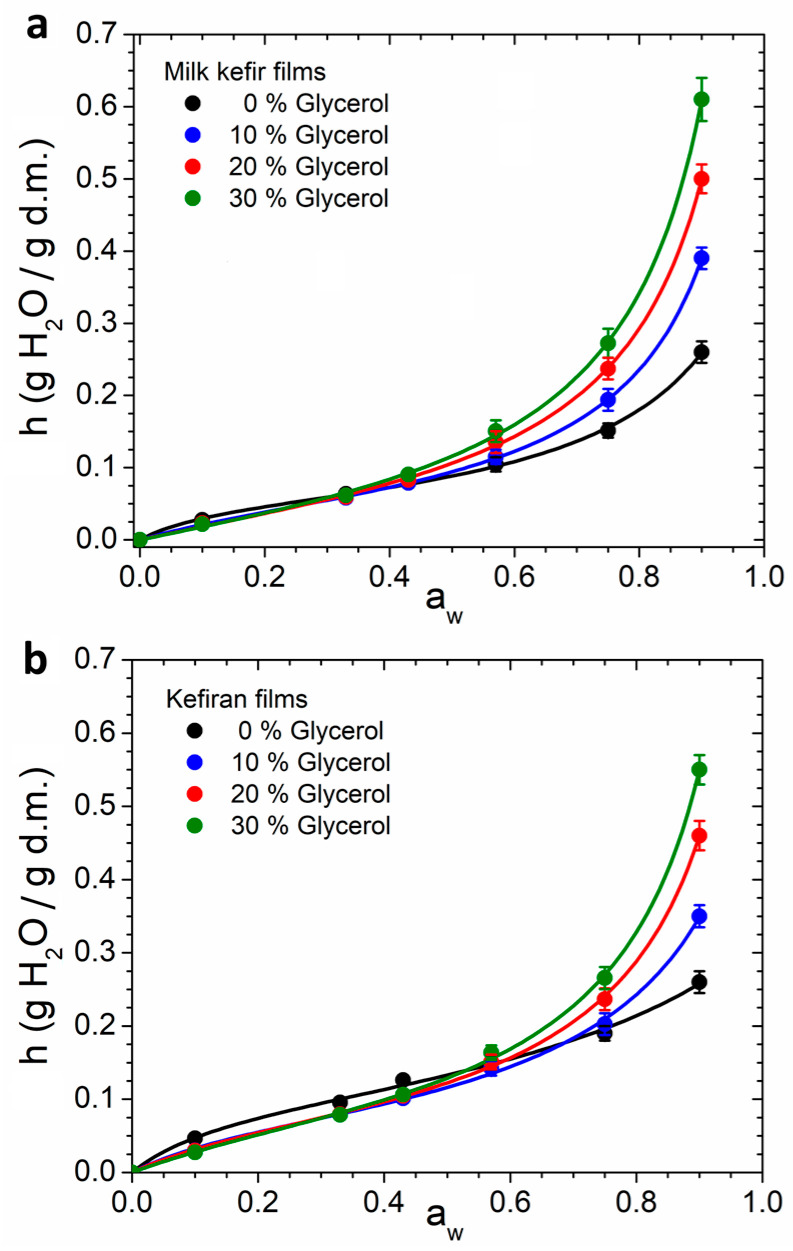
Water vapour sorption isotherms of films with different content of glycerol. (**a**) Milk kefir films. (**b**) Kefiran films. Experimental data were fitted with the GAB model using Equation (2). The fitting parameters are shown in Table 3.

Biopolymer-based films are hydrophilic matrices that are susceptible to moisture uptake, which significantly affects their storage conditions, shelf life, and overall performance. Hydration water plays a crucial role in influencing the structural and functional properties of hydrophilic films. It acts as a plasticiser by inserting itself between the polymer chains, increasing space between them, lowering the glass transition temperature *T*_g_, and enhancing flexibility [7,9]. Water sorption isotherms provide essential insights into the interactions between water and the film, as well as the distribution of water within the material [32,34,60]. Figure 7a,b presents the water sorption isotherms for milk kefir and kefiran films with varying glycerol content. The experimental data were fitted to the GAB model (Equation (4)), with the corresponding parameters summarised in Table 3.

Figure 7a shows that the water sorption isotherms of milk kefir films exhibit a slight increase in hydration water content at low *a_w_* values, followed by a pronounced rise for *a_w_* > 0.6. This pattern is typical for most hydrophilic films made from biopolymers [37,60,61,62]. Similar behaviour was observed in the glycerol-plasticised kefiran films, although it was less prominent in the unplasticised kefiran film (Figure 7b). Unfortunately, no prior studies on sorption isotherms for kefiran films have been found in the literature, with existing research focusing only on the water content of the films after casting [21,22,23,24], which provides limited information on hydration.

As demonstrated in Figure 7a, the addition of glycerol increased the water content *h* for *a_w_* > 0.6, while maintaining the overall shape of the isotherms. This behaviour has also been observed in films based on polysaccharides [62], proteins [61], and natural multicomponent films such as integral water kefir grains biomass [7] and integral cellulosic Kombucha tea by-product biomass [9]. On the other hand, the isotherms for the unplasticised kefiran film were found to be more concave for *a_w_* < 0.6 compared to the glycerol-plasticised films (Figure 7b). A similar behaviour was observed in potato-starch-based films plasticised with varying glycerol concentrations [14].

The observed convexity at *a_w_* > 0.6 in all isotherms in Figure 7 suggests the predominance of multilayer hydration within these films [37]. The increase in hydration as a function of the glycerol content was particularly notable at *a_w_* = 0.9, as evidenced by the measured *h*_90%rh_ values (Table 3). Comparing the *h*_90%rh_ values for unplasticised milk kefir and kefiran films reveals no differences. However, when plasticised with glycerol, milk kefir films show significantly higher *h*_90%rh_ values. This suggests that the interaction of glycerol with proteins in milk kefir films enhances hydrophilicity compared to pure kefiran films plasticised with glycerol.

The hydration equilibrium value at 90% r.h. (*h*_90%rh_) for unplasticised milk kefir and kefiran films was found to be lower than of other unplasticised biopolymeric films, such as cellulose films [63], integral Kombucha tea by-product biomass films [9], starch films [60], myofibrillar protein films [64], and sodium caseinate film [65]. This indicates that films obtained from milk kefir grains and the purified kefiran exhibit less hydrophilicity compared to other biopolymer-based materials. Consistent with these findings, other studies have reported higher water contact angles for kefiran films compared to other polysaccharide-based films, indicating greater surface hydrophobicity and reduced wettability [23].

Table 3 summarises the parameters obtained from fitting sorption isotherms to the GAB model. The parameter *N*, representing the number of primary binding sites of hydration, increased with glycerol content in milk kefir films but decreased in kefiran films. On the other hand, as the glycerol content increased in both milk kefir and kefiran films, the parameter *c*, which reflects the strength of the water binding at primary sites, decreased, while *k*, associated with the capacity of water binding to the multilayers, increased. Consequently, since most of the hydration water forms multilayers, the *h*_90%rh_ values increased with rising glycerol content in both types of films. Glycerol interacts with kefiran and protein chains by forming hydrogen bonds with the reactive groups of these polymers. This disrupts interpolymer interactions, reduces the attractive forces between polymer chains, and increases the free volume available for water molecules, leading to an overall increase in hydration water content.

### 3.8. Water Vapour Permeability of the Films

Hydration water is closely related to the water vapour transport properties of hydrophilic polymeric films [12,37]. The water vapour permeability of biopolymer-based films is a critical property that determines their ability to regulate water vapour transport between a system, such as food, and its environments. SEM studies on milk kefir and kefiran films revealed a continuous and homogenous matrix, without pores, faults, or punctures (Section 3.2) These findings indicate that water transport in milk kefir and kefiran films does not occur through pores, but via a sorption–diffusion–desorption mechanism [39]. Consequently, the water vapour permeability is influenced by the hydration or water solubility within the film, as well as the mobility of water molecules in the matrix [37].

Water vapour permeability in biopolymer films is significantly influenced by experimental conditions, particularly film thickness [12,37] and water vapour pressure gradient [37,39]. To ensure accurate comparisons, all of the films in this study were maintained at a consistent thickness (see Table 4) and identical water vapour pressure gradient conditions. Table 4 presents experimental water vapour permeability values (*P_w_^exp^*) for milk kefir and kefiran films with varying glycerol content. It can be seen that for milk kefir films, *P_w_^exp^* increased with glycerol content. Glycerol addition typically increases water vapour permeability, as observed in various biopolymer-based films, including those made from corn starch [60], myofibrillar protein [64], cellulose [66], β-lactoglobulin [67], water kefir grains biomass [7], and yeast biomass [12]. It has been suggested that the addition of glycerol increases the permeability of the films mainly due to the increase in hydration and solubility of water within the material [12]. Interestingly, as shown in Table 4, unplasticised kefiran films exhibited the highest *P_w_^exp^* values among all kefiran samples. Kefiran films containing 10 wt% glycerol presented the lowest *P_w_^exp^* values. Subsequent glycerol additions led to increased *P_w_^exp^*, although values remained below those of the unplasticised film.

Table 4 additionally presents *S_w_^eff^* and *D_w_^eff^* values, which independently contribute to *P_w_^exp^*, as described in Equation (6). Both sample types exhibited a significant increase in water solubility *S_w_^eff^* and a decrease in water diffusion *D_w_^eff^* with rising glycerol content. Milk kefir films demonstrated a significantly greater increase in *S_w_^eff^* compared to the decrease in *D_w_^eff^* with increasing glycerol content, resulting in an overall increase in *P_w_^exp^*. On the other hand, *D_w_^eff^* for the unplasticised kefiran film was found to be remarkably high compared to the kefiran film plasticised with 10 wt% glycerol. However, with the addition of more glycerol, the *D_w_^eff^* value decreased only slightly, similar to what was observed in milk kefir samples. As a result, the unplasticised kefiran film exhibited the highest *P_w_^exp^* value, while the film with 10 wt% glycerol had the lowest. With each subsequent addition of glycerol, *P_w_^exp^* increased; however, it never reached the level observed in the absence of the plasticiser.

This anomalous effect of glycerol on the water vapour permeability of kefiran films was also observed by Piermaria et al. [22], who attributed this behaviour to the development of a more compact structure in plasticised films. As shown in Table 4, the density values (*ρ_d.f._*) of milk kefir and kefiran films exhibit a slight tendency to increase with the glycerol content; however, the anomaly was observed only in the kefiran film. For kefiran films, this anomaly could be the result of a stronger interaction between glycerol, water molecules, and the available (–OH) groups of kefiran. Given the structure of the kefiran film, these components can engage in multiple interactions via hydrogen bonds, thereby retarding water movement. Once the (–OH) groups of kefiran are saturated at 10 wt% glycerol, the addition of more plasticiser leads to a smaller decrease in *D_w_^eff^*, while *S_w_^eff^* increases notably. Consequently, the *P_w_^exp^* of kefiran film increased but did not reach the level observed in the absence of plasticiser. In contrast, in milk kefir films, the protein–kefiran matrix may inhibits these specific interactions between glycerol, water molecules, and the available (–OH) groups of the composite matrix. As a result, the anomalous behaviour observed in kefiran films is not present in this sample.

Furthermore, the anomalous effect of glycerol on water vapour permeability was also observed in potato-starch-based films, which exhibited water sorption isotherm behaviour at varying glycerol concentrations similar to that of kefiran films [14]. It has been suggested that plasticisers may either retard or facilitate moisture transmission depending on their concentration [63]. The decrease in *P_w_^exp^* to a minimum value at low glycerol concentration, followed by an increase at higher plasticiser levels, can be attributed to the antiplasticisation effect at low plasticiser concentration described in Section 3.6. This effect arises from a specific interaction between the polymer and the plasticiser molecules, which reduces the molecular mobility of the polymer chains, as was observed in the mechanical properties of kefiran films (Section 3.6). A similar antiplasticisation effect has been observed in the mechanical behaviour and water permeability of cellulose acetate films [13].

Although the precise mechanism behind antiplasticisation at low plasticiser concentrations remains unclear, one hypothesis suggests that incorporating small amounts of plasticiser into a polymer increases the free volume, allowing for the polymer chains to rearrange into a more thermodynamically stable and compact structure [13]. Unfortunately, the scanning electron microscopy images obtained in the present study do not offer sufficient resolution to observe these structural changes. Further characterisation of these polymeric structures is required, as it could enhance our understanding of plasticisation and antiplasticisation phenomena, which would be of significant interest to the field of materials science. In conclusion, the observed *P_w_^exp^* behaviour as a function of the glycerol content in kefiran films is likely the result of structural modifications within the kefiran matrix induced by the glycerol, which restrict water transport at low concentrations.

## 4. Conclusions

Films derived from both the integral biomass of the water kefir grains and from the purified kefiran exhibited excellent continuity and homogeneity. While kefiran films were transparent, milk kefir films had a yellowish tint. The application of ultrasonic treatments to both film-forming dispersions allowed for the use of 5 wt% d.m. concentration, reducing the energy required for drying during the casting process.

Kefiran films showed stronger interpolymeric interactions compared to milk kefir films, as evidenced by thermogravimetric analyses and mechanical testing. These findings suggest that the presence of proteins in the milk kefir matrix weakens interpolymeric interactions. Glycerol increased ductility and hydration while decreasing the thermal stability and glass transition temperature of the films.

Additionally, kefiran films exhibited an antiplasticisation effect at low glycerol content, affecting elongation at break and water vapour permeability. This unexpected behaviour suggests specific characteristics in the interactions between glycerol and kefiran. The absence of this effect in milk kefir films indicates that the presence of proteins inhibits it.

Traditionally, biopolymeric materials are developed by purifying biopolymers from their original biomass and subsequently modifying them physically or chemically to enhance their film-forming properties. However, the results of this study highlight the potential for using integral milk kefir grains directly in the development of novel biodegradable materials. These findings emphasise the differences between materials derived from the integral milk kefir grains and those from purified kefiran, providing insights into their application potential.

The hydrophilicity and sensitivity to high humidity of these materials make them most suitable for food packaging applications involving dry or fatty foods. However, with further experimentation, this hydrophilicity could be exploited to develop delivery packaging for instant products, where the packaging dissolves in hot water and potentially modifies the rheology of the final product. While these materials may not fully replace petroleum-based polymers, they provide a viable alternative for specific applications, supporting a shift toward a more sustainable and circular economy.

## Figures and Tables

**Table 1:** Parameters obtained from fitting the Herschel–Bulkley model (Equation (1)) to the flow curves of Figure 1. MK denotes milk kefir and KEF. Errors in the parameters were estimated from the fit analysis.

*Sample*	*τ*_0_ (10^−3^ Pa)	*K* (10^−3^ Pa·s)	*n*	*R* ^2^
MK 0% Glycerol	42 ± 2	10.9 ± 0.2	0.916 ± 0.002	0.9999
MK 10% Glycerol	32 ± 2	9.6 ± 0.2	0.928 ± 0.003	0.9999
MK 20% Glycerol	33 ± 2	9.4 ± 0.2	0.932 ± 0.003	0.9999
MK 30% Glycerol	30 ± 2	9.3 ± 0.2	0.933 ± 0.003	0.9999
KEF 0% Glycerol	54 ± 3	34.8 ± 0.2	0.988 ± 0.001	0.9999
KEF 10% Glycerol	84 ± 5	36.3 ± 0.3	0.972 ± 0.001	0.9999
KEF 20% Glycerol	73 ± 4	34.8 ± 0.2	0.980 ± 0.001	0.9999
KEF 30% Glycerol	79 ± 4	34.7 ± 0.2	0.987 ± 0.001	0.9999

**Table 2:** Mechanical parameters of milk kefir films (MK) and kefiran films (KEF). The mean and standard deviation are reported. The different letters assigned in each column refer to significant differences (*p* ≤ 0.05). n.d. denotes not determined.

*Sample*	*Y (MPa)*	*TS_max_ (MPa)*	*e_%_ (%)*
MK 0% Glycerol	n.d.	n.d.	n.d.
MK 10% Glycerol	443 ± 33 ^b^	14 ± 2 ^c^	4 ± 1 ^f^
MK 20% Glycerol	155 ± 19 ^d^	7 ± 1 ^d^	20 ± 4 ^c^
MK 30% Glycerol	63 ± 9 ^e^	4 ± 1 ^e^	34 ± 3 ^b^
KEF 0% Glycerol	567 ± 61 ^a^	34 ± 3 ^a^	13 ± 2 ^d^
KEF 10% Glycerol	544 ± 48 ^a^	20 ± 4 ^b^	8 ± 2 ^e^
KEF 20% Glycerol	313 ± 25 ^c^	9 ± 2 ^d^	38 ± 8 ^b^
KEF 30% Glycerol	146 ± 21 ^d^	5 ± 1 ^e^	72 ± 10 ^a^

**Table 3:** Parameters obtained from fitting the GAB model (Equation (2)) to the water sorption isotherms of Figure 7. Errors in the GAB parameters were estimated from the fit analysis. *h*_90%rh_ refers to hydration equilibrium values measured at 90% r.h. MK denotes milk kefir and KEF. The different letters assigned to each column refer to significant differences (*p* ≤ 0.05).

*Sample*	*h_90%rh_* (g·g^−1^)	*GAB Parameters*			
		*N* (g·g^−1^)	*c*	*k*	*R* ^2^
MK 0% Glycerol	0.26 ± 0.01 ^a^	0.056 ± 0.002	9.6 ± 0.9	0.876 ± 0.009	0.999
MK 10% Glycerol	0.39 ± 0.01 ^c^	0.064 ± 0.001	4.2 ± 0.4	0.937 ± 0.003	0.999
MK 20% Glycerol	0.50 ± 0.02 ^e^	0.082 ± 0.004	2.5 ± 0.4	0.941 ± 0.007	0.999
MK 30% Glycerol	0.61 ± 0.03 ^g^	0.094 ± 0.005	2.0 ± 0.3	0.954 ± 0.006	0.999
KEF 0% Glycerol	0.26 ± 0.01 ^a^	0.104 ± 0.009	10 ± 1	0.690 ± 0.008	0.996
KEF 10% Glycerol	0.35 ± 0.01 ^b^	0.079 ± 0.005	6.5 ± 0.7	0.871 ± 0.007	0.998
KEF 20% Glycerol	0.46 ± 0.01 ^d^	0.080 ± 0.003	5.4 ± 0.5	0.925 ± 0.006	0.999
KEF 30% Glycerol	0.55 ± 0.02 ^f^	0.088 ± 0.004	3.9 ±0.5	0.941 ± 0.007	0.999

**Table 4:** Experimental water vapour permeability (*P_w_^exp^*), water solubility (*S_w_^eff^*), and water diffusion (*D_w_^eff^*). *L* and *ρ_d.f._* refer to the thickness and density measurements of the dried film, respectively. MK denotes milk kefir and KEF. The same letters in the data reported in a column mean non-significant differences (*p* < 0.05).

*Sample*	*L* *(10* ^−5^ *g m)*	*ρ_d.f_* *(10* ^4^ *g m* ^3^ *)*	*P_w_^exp^* *(10* ^−10^ * g s* ^−1^ *m* ^−1^ *Pa* ^−1^ *)*	*S_w_* *(g m* ^−3^ *Pa* ^−1^ *)*	*D_w_* *(10* ^−13^ *m* ^2^ *s* ^−1^ *)*
MK 0% Glycerol	15.6 ± 0.7 ^ab^	113 ± 9 ^ab^	3.2 ± 0.1 ^a^	124 ± 6 ^b^	26 ± 2 ^d^
MK 10% Glycerol	15.1 ± 0.8 ^ab^	121 ± 10 ^ab^	4.6 ± 0.1 ^c^	211 ± 10 ^d^	22 ± 1 ^c^
MK 20% Glycerol	14.5 ± 0.8 ^ab^	131 ± 9 ^ab^	5.6 ± 0.1 ^e^	295 ± 15 ^f^	19 ±1 ^b^
MK 30% Glycerol	14.1 ± 0.7 ^b^	139 ± 9 ^b^	6.1 ± 0.1 ^f^	388 ± 19 ^g^	16 ± 1 ^a^
KEF 0% Glycerol	16.0 ± 0.7 ^a^	104 ± 9 ^a^	5.0 ± 0.1 ^d^	105 ± 8 ^a^	48 ± 4 ^f^
KEF 10% Glycerol	15.4 ± 0.8 ^ab^	113 ± 10 ^ab^	4.0 ± 0.1 ^b^	172 ± 10 ^c^	23 ± 2 ^cd^
KEF 20% Glycerol	14.9 ± 0.8 ^ab^	121 ± 10 ^ab^	4.7 ± 0.1 ^c^	247 ± 11 ^e^	19 ± 1 ^b^
KEF 30% Glycerol	14.3 ± 0.8 ^b^	129 ± 9 ^b^	4.8 ± 0.1 ^cd^	321 ± 12 ^f^	15 ± 1 ^a^

## Data Availability

The data presented in this study are available on request from the corresponding author.
